# Adequacy Assessment of a Universal Salt Iodization Program Two Decades after Its Implementation: A National Cross-Sectional Study of Iodine Status among School-Age Children in Tunisia

**DOI:** 10.3390/nu9010006

**Published:** 2016-12-25

**Authors:** Radhouene Doggui, Myriam El Ati-Hellal, Pierre Traissac, Lilia Lahmar, Jalila El Ati

**Affiliations:** 1 INNTA (National Institute of Nutrition and Food Technology), SURVEN (Nutrition Surveillance and Epidemiology in Tunisia) Research Laboratory, 11 Rue Jebel Lakhdar, babSaadoun, Tunis 1007, Tunisia; jalila.elati@rns.tn; 2 Institut Préparatoire aux Etudes Scientifiques et Techniques, Toxicology Research and Environment Research Laboratory, 10, Rue Abou El KacemChabbi, Montfleury, Tunis 1008, Tunisia; mfh22002@yahoo.fr; 3 IRD (Institut de Recherche pour le Développement), NUTRIPASS Unit, IRD-UM-SupAgro, 911, Av Agropolis, 534394 Montpellier CEDEX, France; pierre.traissac@ird.fr; 4 Hôpital d’Enfants, Pediatric Radiology Department, Bab Saadoun, Tunis 1007, Tunisia; lilialahmar@hotmail.com

**Keywords:** universal salt iodization, iodine deficiency disorders, iodine status, Tunisia, children, adequacy assessment, evaluation

## Abstract

In the framework of a worldwide policy to eliminate iodine deficiency (ID) disorders, universal salt iodization was adopted in Tunisia two decades ago. The present study aims to evaluate this strategy, using both performance and impact indicators. A total of 1560 children, aged 6–12 years, were randomly sampled using a national, two-stage, stratified, cross-sectional cluster survey in 2012. Urinary iodine concentration (UIC) of the subjects, and household salt iodine content, were analyzed. The national median UIC was 220 µg/L (95% confidence interval (CI): 199–241), indicating an acceptable iodine status at the population level. Only 11.4% (95% CI: 8.6–14.9) of the children had UIC <100 µg/L, but with large regional disparities (4.3% to 25.5%, *p* < 0.01); however, more than a quarter of the subjects were at risk of adverse health consequences due to iodine excess. Children from households of low socio-economic levels were more prone to inadequate UIC. The national median iodine concentration of household salt was 22 mg/kg (95% CI: 21–23). Only half of the households used adequately iodized salt (15–25 ppm), with large regional disparities. National ID rates are now well below the target criteria of WHO (World Health Organization) certification (<20% of children with UIC <50 µg/L and <50% with UIC <100 µg/L). The coverage of adequately iodized salt fell short in meeting the goals of USI (Universal Salt Iodization), i.e., >90% of households. Regular monitoring of iodized salt production lines must be strengthened with involvement by producers.

## 1. Introduction

Micronutrient deficiency is a silent worldwide epidemic that impacts morbidity, mortality, and quality of life, and impairs socio-economic development, especially in low- and middle-income countries [1]. Among micronutrients, iodine is essential for thyroid function and the synthesis of thyroid hormones, which are implicated in several metabolic pathways, including nervous system maturation and growth [2]. Inadequate iodine intake may result in a variety of disorders, termed iodine deficiency disorders (IDD), such as goiter, cretinism, spontaneous abortion, perinatal mortality, and heart failure [2]. The occurrence of chronic iodine deficiency (ID) during pregnancy causes hypothyroidism, which is detrimental to the neurological fetal development and, thus, mental retardation. During childhood and adolescence, ID may impair physical and cognitive function development [2]. On the other hand, excess of iodine may also impair thyroid function [3].

It is estimated that 1.9 billion people have insufficient ID intake (data from 2012), of which 246 million are school-aged children [2]. To optimize iodine intake, universal salt iodization (USI) was adopted as a worldwide strategy as of 1994. As part of their public health policies, a number of countries have implemented USI programs, of which the efficiency in reducing or eliminating ID needs to be monitored using relevant performance and impact indicators [4,5]. Although ID is a worldwide issue, some regions are especially affected by this micronutrient deficiency, in particular the Middle East and North Africa (MENA): Countries, such as Algeria and Morocco, are within the top ten countries most affected by IDD [2,6,7]. Tunisia is a typical middle-income country in the MENA region, having undergone a rapid socio-economic development and nutrition transition in the last few decades: The associated lifestyle changes, including “Westernization” of diet, have increased energy, sugar, fat, and salt intakes [8,9]. These have contributed to high prevalences of non-communicable diseases, such as obesity, type 2 diabetes, or hypertension, which co-exist with still-prevalent conditions linked to micronutrient deficiencies, e.g., anemia [10,11,12].

With respect to IDD, in 1973–1975, a national survey underlined the northwest of Tunisia as a goiter endemic region, as the prevalence of goiter was three times higher in this region compared to the others (9.3% vs. 3.3%), especially in women of reproductive age (23% vs. 11%, respectively) [13]. Mandatory legislation on salt iodization was launched in 1984 in IDD areas [14]. A second national survey in 1995, conducted among children aged 8–11 years showed that this action was not sufficient [15]. Thus, the USI program was adopted in 1995 [16] and implemented in 1996 [17], led by the ministry of health and supported mainly by the UNICEF.

In 2000, based on criteria pertaining only to salt iodine content, Tunisia was declared IDD free [18]. However, since 2011, Tunisia has aimed at obtaining the “sustainable elimination of ID” certification from WHO, which includes criteria pertaining to both salt iodine content and UIC [4]. Thus, the objectives of the present study were to assess: (i) the adequacy of the Tunisian strategy for improving iodine status using both process (quality of households iodized salt) and impact (UIC among school-age children) indicators [5]; and (ii) how these indicators varied according to place of residence, socio-economic characteristics, and individual characteristics.

## 2. Experimental Section

### 2.1. Study Area

Tunisia is a North African country, with a population of about eleven million inhabitants [19]. It features a middle income level of development [20], which is unevenly distributed across the seven administrative regions. The level of economic development is higher in the northern and eastern coastal regions, but the mountainous western inland parts, especially the north- and central-west regions, or the mainly desert south-west, feature a much lower level of economic development.

### 2.2. Design and Subjects

The design aimed at an adequacy assessment evaluation of the Tunisian national salt iodization program, based on a national cross-sectional study performed during May and June, 2012 [5]. According to recommendations for the monitoring of iodine status in populations, the target population was Tunisian children aged 6–12 years [4]. The source population was children aged 6–12 years attending public or private schools: It was stratified according to the 24 governorates (administrative divisions), which compose Tunisia and the living area (urban/rural). A two stage cluster sampling was performed [21]: First, by systematic random sampling using a list of primary schools obtained from the Ministry of Education; two primary-schools were selected in each strata (three for the governorates of the north-west region, for which specific estimates were needed as it was historically prone to IDD [22]; in the second stage, 30 eligible children (15 boys and 15 girls) were randomly sampled in each selected school. In each school, a list of randomly selected replacement subjects was established in order to minimize bias due to non-response (e.g., refusals or absence). The expected sample size was then a total of 52 × 30 = 1560 children.

### 2.3. Socio-Economic and Anthropometric Assessment

All data and measures were collected by trained field agents. Data on level of education and occupation of children’s parents were collected using an auto-administered questionnaire, sent to them few days before the survey, and returned by the children the day of the survey. With respect to anthropometry, standing height was measured to the nearest 0.1 cm with the use of a wall-mounted stadiometer (Person-check^®^, Kirchner and Wilhelm, Germany); weight was measured to the nearest 0.1 kg on a calibrated scale (Detecto, Webb City, MO, USA). Height-for-age and BMI (Body Mass Index = weight/height^2^) for-age *z*-scores were derived from the World Health Organization (WHO) reference for school-age children: Stunting was defined as height-for-age <−2 z, thinness was defined as BMI-for-age <−2 z, and overweight as BMI-for-age ≥+1 z [23].

### 2.4. Urinary Iodine Concentration

**Figure A1:**
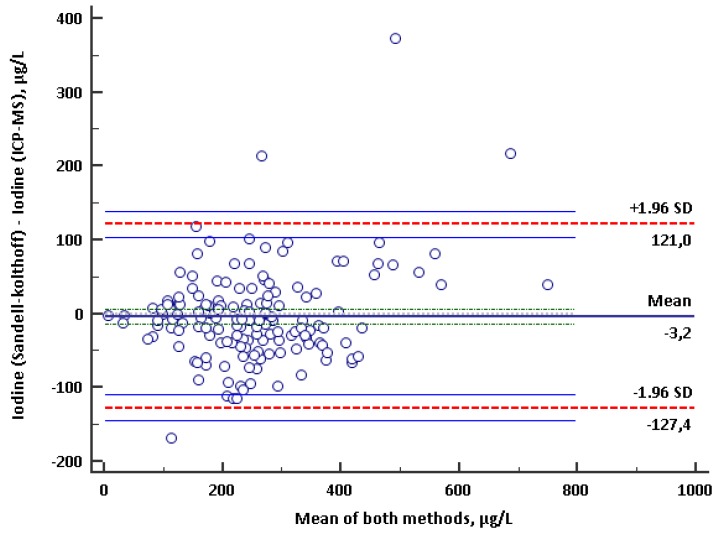
Bland–Altman analysis of urinary iodine measurement (µg/L) by ICP-MS and Sandell–kolthoff. The scatter diagram plots the difference of values against the averages of values of the two methods.

A casual urine sample (20 mL) was collected in clean and sterile tightly capped vials, which were pre-labeled. Special precautions were taken to avoid sample contamination. The enumerators were instructed to ensure that every child went to the toilet individually. Furthermore, special precautions were undertaken in order to maintain the cold chain during the transportation of the samples. All samples were kept at 4–5 °C and were sent to the Laboratory of Clinical Biology at the National Institute of Nutrition and Food Technology within 12 h. Samples were aliquoted and stored at −20 °C until the day of analyses. UIC was measured using the Sandell-kholtoff reaction [4]. Commercial control material was included in each batch of samples (Seronorm Trace Elements Urine L-2, REF 110412, lot 1011645). The coefficient of variability was 7.1% and the limit of quantification was 17.26 µg/L. External quality controls were provided by the Biochemistry, Toxicology and Pharmacology Laboratory, of CHU-Grenoble (Centre Hospitalo-universitaire de Grenoble), France, which uses ICP-MS ((Inductively Coupled Plasma), which considered as a reference method for UIC quantification: A correlation coefficient of 0.874 and a Bland and Altman plot (Figure A1 in Appendix A) [24] confirmed the agreement between the two methods.

Interpretation of iodine status at a population level, followed the WHO criteria based on median UIC in µg/L, which defined seven degrees of public health significance [4]: Severe ID if <20, moderate ID if ≥20 and <50, mild ID if ≥50 and <100, optimal iodine status if ≥100 and <200, risk of iodine-induced hyperthyroidism if ≥200 and <300, excess of iodine with risk of adverse health consequences if ≥300 and <500, excess of iodine with adverse effects of chronic iodine excess if ≥500). We also took into account the recent recommendation of the WHO/IGN (Iodine Global Network), which indicated that the acceptable range of median UIC in monitoring iodized salt programs could be widened to a single category of sufficient iodine intake from ≥100 to <300 µg/L [4]. At the subject level, due to a lack of internationally acknowledged cut-offs for iodine status of individuals, and for analyses purposes, we also used the same cut-offs, as presented above, for the population level, to categorize iodine status of subjects according to a 7 or a 3 category variable (<100, ≥100 to <300, ≥300).

### 2.5. Iodine Content of the Salt Used in Households

Each child was instructed to bring a salt sample from home (approximately 30 g), in self-sealing polythene bags provided by the field team, to school on the day of the survey. All samples were sent to the National Institute of Nutrition and Food Technology of Tunis, where iodine content was quantified using the titration method [25]. For ease in comparing with international standards, categorization of iodine content was based on both international and national recommendations. According to international recommendations, salt was considered adequately iodized when the iodine concentration in mg/kg (i.e., 1 mg potassium iodate = 1 mg iodine × 1.685) was 15 to <25 (15 to 27 according Tunisian legislation), non-iodized when concentration was null, inadequately iodized for non-zero concentration but <15, and excessively for ≥25 (≥27 in accordance with Tunisian legislation).

### 2.6. Data Management and Statistical Analyses

Epidata software version 3.1 (The Epidata Association, Odense, Denmark, 2008) was used for data entry and validation by double entry and Stata 14 (Stata Corporation, College Station, TX, USA, 2015) for data management and statistical analyses. The type I error risk was 0.05. Results are presented as estimates and standard error (between parentheses) or a 0.95 confidence interval (between brackets).

For the UIC and iodine content of the salt used in the households, results were expressed as medians and/or percentages using the categorizations defined above. Associations between UIC or salt iodine content with gender, age, and area or region, were graphed using box and whisker plots of quantitative variables and assessed using chi-square tests, using UIC or salt iodine content in quintiles. At the regional level, an ecological analysis assessed the relationship between median salt iodization and median UIC. One way Anova performed on the log transformation of UIC was used to compare geometric means of UIC between categories of salt iodization (<15, ≥15 to <25, ≥25). Multinomial logistic regression was used to assess crude and adjusted associations of the subject-level UIC in 3 categories (<100, ≥100 to <300, ≥300 µg/L) with living area, region, gender, age, education, and occupation of the father and the mother, BMI-for-age, and iodine content of salt used in the household. These models, fitted with the Stata *mlogit* command, used the ≥100 to <300 µg/L category as the base response category, and associations were quantified as crude of adjusted Relative Prevalence Ratios (RPR) [26].

All analyses took into account the sampling design (stratification, clustering, sampling weights accounting for unequal probabilities of selection) using *svy* Stata commands, specific to survey data analysis [27]. Due to the lack of specific *svy* Stata commands for estimation of medians from complex survey sampling plans, weighted median estimates, and robust confidence intervals were derived using the *qreg* (quantile regression) Stata command. The design effect, due to the cluster sampling, was then estimated from comparing weighted vs. design-based estimates of the means. The design effect was then applied to derive design-based standard errors and/or confidence intervals for the medians.

### 2.7. Ethical Approval

All applicable institutional and governmental regulations concerning the ethical use of human volunteers were respected during this study. The protocol of the survey was reviewed and approved by the Ethics Committee on Human Research of the National Institute of Nutrition and Food Technology, and the Tunisian National Council of Statistics (visa n° 8/2012). After being thoroughly informed on purpose, requirement, and procedures, all parents of the children included in the survey gave their free informed consent.

## 3. Results

### 3.1. General Characteristics of the Samples

A total of 780 boys and 780 girls were surveyed, from which urine and salt samples, and socio-demographic and anthropometric data were collected. Two thirds were from urban areas and weighted proportions by regions were in accordance with national data for the same age-class (Table 1). Mean age was 9.3 (0.04) years, the minimum was 6.1 years and maximum was 12.9 years in accordance with the inclusion criteria. Most fathers and mothers had primary schooling or more, but seven mothers out of 10 had no professional occupation vs. only a fraction of the fathers. Mean weight was 30.5 (0.5) kg, mean height 134.3 (0.5) cm, mean height-for-age was 0.0 (0.1) *z*-scores, mean BMI-for-age was −0.18 *z*-scores of the reference. Less than a 10th of the children were thin. About one out of five was overweight, more girls than boys were overweight.

### 3.2. Urinary Iodine Concentration

*National.* The distribution of UIC was somewhat asymmetric with a mild skewing to the right with a median of 220 µg/L (95% CI: 199–241) denoting a trend towards normal to high levels of urinary iodine. Values were dispersed within a wide range, from 3 to 991 µg/L, and the interquartile range was 146 to 300 µg/L. The prevalence of UIC < 100 µg/L was 11.4% (95% CI: 8.6–14.9), 3.1% (95% CI: 2.0–4.7) had UIC < 50 µg/L, and only 0.4% (95% CI: 0.2–0.8) had UIC < 20 µg/L. At other extremes, 25.1% (95% CI: 19.6–31.4) had high UIC levels (≥300 µg/L) and 4.2% (95% CI: 3.0–5.7) had very high UIC levels (≥500 µg/L). Hence 63.5% (95% CI: 57.7%–69.0) of the subjects had UICs in the ≥100 to <300 µg/L iodine range and 32.3% (95% CI: 28.1–36.9) were in the optimal ≥100 to <200 µg/L range.

*By gender and age* (Table 2). Distribution of UIC was shifted towards higher values for boys vs. girls (*p* = 0.014) with medians, respectively, of 241 (13) µg/L and 200 (9) µg/L; there were no marked gender differences in prevalence of UIC within 50–99 µg/L (9.9% vs. 13.1%, respectively) and UIC < 50 µg/L (2.1% vs. 3.3%) but girls were much less prone than boys to UIC levels ≥300 µg/L (19.6% vs. 30.1%, *p*< 0.001). There were no marked differences in median UIC values according to age. Nevertheless, due to a trend in decreasing variability of UIC with increasing age, the prevalence of UIC < 100 µg/L somewhat decreased with age (from 13.4% for children aged 6–9 years to 8.7% among 9–12 years, *p* = 0.085). This also was observed for UIC < 50 µg/L prevalence (3.7% for 6–9 years to 2.1% for 9–12 years).

*By place of residence* (Table 2). There was no difference in the distribution of UIC between urban and rural areas. Within both urban and rural areas, boys had higher UIC than girls, as observed nationally. There were very large differences between regions regarding UIC distribution (*p*< 0.0001): Medians ranged from a low of 159 µg/L in the north-east, to values as high as 271 µg/L in the south-west, or even superior to the acceptable 300 µg/L threshold in the south-east at 339 µg/L. Thus the prevalence of UIC < 100 µg/L varied markedly between regions, from 4.3% (0.8% with UIC < 50 µg/L) in the south-west and 7.5% (2.6% with UIC < 50 µg/L) in the south-east to a high of 25.5% (6.1% with UIC < 50 µg/L) in the north-east (*p* = 0.009). Conversely, prevalence of UIC ≥ 300 µg/L varied from a low of 15.2% in the central-east and a high of 34.2% in the south-west, and a staggering 62.5% in the south-east (*p* < 0.001). Difference between genders within regions was generally similar to those observed nationally.

### 3.3. Iodine Content of Salt Used in Households

**Figure 1:**
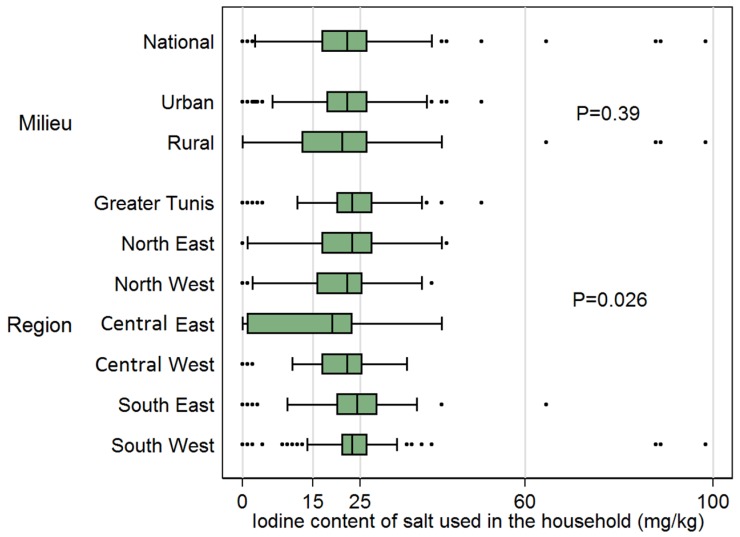
Iodine content of salt used in the households of Tunisian children aged 6–12 years. P-values: design based chi-square test for salt iodine content in quintiles (as figured by vertical reference lines) × factor variable.

At the national level, the iodine content of the salt samples from the 1560 households featured a median of 22 mg/kg (95% CI: 21–23), within the target range, but also a large variability (Figure 1). According international standards, 6.2% used non-iodized salt, 15.6% inadequately iodized salt, and 34.1% excessively iodized salt, and only 44.1% was adequately iodized (respectively, 6.2%, 15.6%, 22.4%, and 55.8%, in accordance with Tunisian legislation). Distribution of iodine salt content was similar in rural and urban areas (medians, respectively, of 22 mg/kg and 21 mg/kg). Though all medians were within the target range, the distribution of salt iodine content varied with region (*p* = 0.026), with medians ranging from 19 mg/kg in the central-east to 24 mg/kg in the south-east. Due to the difference between medians and differences in variability within regions, there were marked differences in the proportion of non-iodized or inadequately iodized salt (from 24.3% in the central-east to 6.1% in the south-east) or of excessive iodization (from 20.9% in the central-east to 45.4% in the south-east).

### 3.4. Relation between Median Urinary Iodine Concentration and Median Salt Iodine Content at a Regional Level

**Figure 2:**
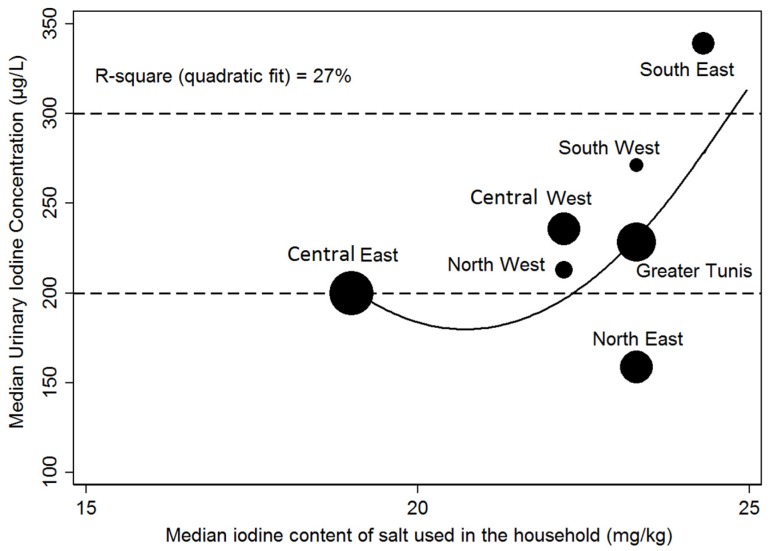
Relationship at the regional level between median salt iodine content and median urinary iodine concentration.

The ecological analysis at the regional level was presented in Figure 2, and it showed that there was a coherent increase of median UIC with median salt iodine content: Quadratic polynomial, R-square = 0.27 (nevertheless in the north-east region median UIC was the lowest despite median salt iodine content being in the upper range of the recommended iodization). There was a significant residual variability of median UIC for a given salt iodine median, especially in the upper ranges of salt iodine content.

### 3.5. Relationship between Subject-Level Iodine Status and Living Environment, Socio-Demographic Factors, Anthropometry and Salt Iodine Content

The adjusted associations of iodine status (in three categories ID: <100, 100 to <300, ≥300 µg/L) estimated using multinomial regression (Table 3) showed that girls were less prone to iodine excess than boys (*p* = 0.001, RPR = 0.5 (95% CI: 0.4–0.7)) with no difference for UIC < 100 µg/L. Prevalence of UIC < 100 µg/L tended to be lower for the older children (*p* = 0.048). There was no adjusted difference of iodine status between urban and rural areas. The marked unadjusted differences between regions for prevalence of both UIC < 100 µg/L and UIC ≥ 300 µg/L (*p* = 0.0094 and *p* = 0.001) were minimally modified by adjustment (*p* = 0.006 and *p*< 0.001): In particular, the north-east region was the more affected by low iodine status (adjusted RPR = 2.1 (95% CI: 1.4–3.1) vs. Greater Tunis), while children from the south-east region were strikingly prone to excess iodine status (adjusted RPR = 4.9 (95% CI: 1.6–14.7)). Association of UIC with socio-economic factors was generally weak, except that children with fathers with lower levels of education were more prone to excess iodine status (e.g., no schooling vs. secondary or more, adjusted RPR = 1.8 (95% CI: 1.2–2.7)). Additionally, children with fathers in the lower categories of occupation tended to be more prone to low UIC: Not working and employee/worker vs. middle/upper executive, respectively, RPR = 1.9 (95% CI: 1.0–3.5) and 1.7 (95% CI: 1.1–2.5) (Table 3).

There was no straightforward association of UIC < 100 µg/L with BMI-for-age, though more corpulent subjects were marginally more prone to have UIC ≥ 300 µg/L vs. those of normal weights (RPR = 1.4 (95% CI: 0.9–2.1)). The association of household salt iodine content with iodine status was marked for excess iodine. Children of households using non-iodized salt (adjusted *p* = 0.003: RPR = 0.5 (95% CI: 0.2–1.0)) were protected against abnormally-high iodine status. However, there was no observed association of salt iodine content with low UIC.

Medians of UIC in categories of salt iodization (non or inadequately iodized, adequately iodized and excessively iodized) were, respectively, 190 (11) µg/L, 228 (13) µg/L, and 231 (11) µg/L. Nevertheless, the one way Anova performed on log transformed UIC values showed no statistically significant differences between geometric means (*p* = 0.27).

## 4. Discussion

Based on a national random sample of more than fifteen hundred school-aged children of both genders, we conducted an adequacy assessment of the Tunisian national salt iodization program, using both impact and performance indicators. The present survey is the first conducted to assess the iodine status of the Tunisian population, almost two decades after the promulgation of compulsory salt iodization legislation by the Tunisian government in 1995, in accordance with UNICEF and WHO 1994 recommendations of USI as the main strategy for elimination of IDD [4].

### 4.1. Median of Urinary Iodine Content at the National Level Is in the Target Range

Our results showed that median UIC of Tunisian school-aged children was 220 µg/L. Thus, the objectives of the program can be considered as met at the national level, with reference to the adequacy criteria of a median in the 100 µg/L to less than 300 µg/L range, which define acceptable iodine status at population level [4]. Additionally, UIC criteria regarding the “IDD free country” certification [4] were met, as only 3.1% of the children had UIC < 50 µg/L (vs. the 20% upper limit) and 11.4% of them had UIC < 100 µg/L (vs. the 50% upper limit). This latter prevalence of insufficient iodine intake was much lower than that observed in the African continent a whole (41.4%) or the East Mediterranean region (48.8%), and is comparable to the low rates observed in the American continent (10.6%) [2]. Comparison with countries in the region is difficult due to a lack of recent, completely comparable national large scale data. However, it would seem that the epidemiological IDD situation is better in Tunisia than in Morocco (mild ID) and Algeria (severe ID), while there is no data for Libya. Additionally, in Egypt, the last national survey, carried out in 2015, reported optimal iodine medium UIC among schoolchildren and 90% of households using adequately iodized salt [28,29]. IDD’s more favorable status in Tunisia could possibly be attributed to its higher level of economic development (e.g., Human Development Index ranking 94th vs. Morocco 130th over 186 countries in 2012) [20], but it is equivalent to that of Algeria (ranked 93rd), where ID status seems much worse. In addition, it has been shown that, although IDD is generally more prevalent in LMIC, developed countries are not immune from IDD, with the example of Australia, and a number of European countries, which in 2003 had high rates of ID, until these were reduced by a variety of measures, including USI [2]. The favorable evolution of the Tunisian epidemiological IDD situation (in 1995 a national study reported a mean UIC of 158 µg/L among children aged 6–11 years) is, thus, likely attributable to the implementation of the salt iodization program following the 1995 and 1996 legislations [15,16,17], more than to the rapid socio-economic development of Tunisia (25% increase in HDI from 1990 to 2012, i.e., one of the largest worldwide).

This is further confirmed by the increase in the program performance indicator: We observed a median iodine content of 22 mg/kg in the salt used in households, in the international target range of 15 to 25 mg/kg vs. observed values in the 3 to 12 mg/kg range in 1995. It could be noted that, in our subject level analysis, we did not observe a straightforward relationship between use of adequately iodized salt and UIC: Methodological limitations, linked to the assessment of both iodine status at subject level and salt iodine content at the household level, could account for that result. In addition, this would be in accordance with a large body of evidence showing that intakes of iodized salt is the main source of iodine intake and linking USI to reduction in the prevalence of ID in a number of countries [2].

### 4.2. Prevalence of Subjects at Risk of Adverse Health Consequence Due to Excess Iodine Intake Is High

Although our estimate of the median UIC among Tunisian school-aged children was in the 100 to <300 µg/L “acceptable range”, it was beyond the 100 to <200 µg/L “optimal range” according to WHO [4]. Elevation of UIC reflects a recent high iodine intake, of which several factors may be sources. As a consequence of the nutritional transition that Tunisia has been experiencing over the last few decades, and associated dietary changes, daily salt intake is high (around 11 g/person/day according a study on Tunisian adolescents in 2005 [9]). This is likely a main factor of the observed medium high UIC. This shift to the right, and the long right tail of the distribution of UIC, resulted in more than a fourth of the subjects being at risk of adverse health consequences due to high iodine intake (UIC ≥ 300 µg/L) and 4.2% had UIC ≥ 500 µg/L. Indeed, excess UIC is associated with increasing thyroid volume and a risk of adverse effects due to chronic iodine excess (hyperthyroidism, thyrotoxicosis, nodular goiter [3]). One of the factors is salt iodine content, and this is also observed in our study, as subjects that are less prone to high iodine levels are those consuming salt with zero or low iodine contents. The median salt iodine content observed in our study was 22 mg/kg and the large variability of iodine salt content at the national level resulted in that 34.1% of households used excessive iodized salt: That is most certainly a factor in the observed elevated prevalences of high or very high UIC values. Apart from salt, either added or in food products, and beyond consumption of foods rich in iodine, such as sea food, meat, dairy products, eggs, or vegetables [30,31], naturally high iodine concentrations in tap water could also be a factor [32]. Indeed, a study reported that iodine concentration in drinking water in Gabès, a city in the south of Tunisia, was about 73 µg/L [33].

### 4.3. Large Geographic Variability of Iodine Status

There was no difference in iodine status between rural and urban areas, which also featured a quite similar median salt iodine content. There were large disparities between regions regarding iodine status, even when adjusting for a number of subject-level socio-economic or individual factors. The two regions of the south featured the highest medians of UIC, with a large proportion of subjects at risk of adverse health consequences due to iodine excess. The mountainous north-west region (which historically was an endemic goiter area, and for which the first Tunisia salt iodization legislation was specifically drafted in 1984 [14,22,34]), featured a median UIC in the recommended bracket (213 µg/L) and a rather low (7.7%) prevalence of UIC < 100 µg/L. Thus, regarding that historical pocket of ID, the USI program can be considered a success. On the contrary, the neighboring north-east, which, beyond its mountainous part, also features a long coastline, was the region with the highest prevalence of ID, as one subject out of four had UIC < 100 µg/L. There was no obvious pattern of association of UIC and/or median salt iodine content with geography (e.g., inland west vs. coastal east regions), nor in levels of economic development.

The large geographic variability of median iodine salt content likely influenced regional differences in UIC status. Indeed, the ecological analysis underlined that salt iodization explained about a third of UIC variability at that aggregated level of analysis (which likely mitigates the limitations of subject-level assessment regarding either UIC or salt iodine content). Our regional level analyses also underlined that, with the exception of the central-east, regions featuring a rather similar median level of salt iodine content (around 24 mg/kg of iodine) could have somewhat different median UIC (from 176 µg/L to 346 µg/L). Thus, beyond salt iodine content, regional variation in other factors could also somewhat impact iodine status, such as level of salt intake, consumption of sea food, meat, dairy products or eggs, or iodine in tap water [9,31,33]. In addition, the southern part of Tunisia is characterized by an arid climate, where water scarcity is frequently reported [35], thus, irrigated agriculture often has to rely on saline water. This water is iodine-rich [36], thus, food products may concentrate important amounts of iodine [37], and also iodine concentrations in soil and ground water may occur.

### 4.4. Association of Iodine Status with Socio-Economic and Individual Characteristics

As has been observed in some contexts [38], we observed gender differences in iodine status as, although both UIC medians were well within the 100 to <300 µg/L, it was 50 µg/L lower for girls vs. boys. This should warrant special attention in the long term, as ID is especially critical during pregnancy, but also for reasons of gender equity with regards to the importance of iodine status in cognitive development [2]. On the other hand, in a context of high prevalence of iodine excess, girls were less at risk than boys.

In the adjusted analyses, children from lower socio-economic level households were more at risk of inadequate UIC status, either below or above the recommended range. This would seem to be linked to higher variability of salt iodine content in these lower categories, rather than differences in median salt iodine content. In the same context, it has been shown that the type of retail store used for food shopping differs according to socio-economic status. Thus, it should be among the objectives of the USI program that iodized salt of identical and regular quality be available from all retailers, including those used by the lower socio-economic status families.

In the context of the nutritional transition that Tunisia is experiencing, the issue of the possible association between excess adiposity and ID is of importance. However, contrary to what is observed for other micronutrients (e.g., iron [39]) or in some contexts for iodine [40], we did not find such an association.

### 4.5. Large Variability of Iodine Content of Salt Used in Households

In Tunisia, after the promulgation of the 1995 legislation, the decree of September 1995 and the decree of April 1996 fixed the characteristics of iodized salt, its mode of distribution and quality control processes at the level of the entire distribution chain [16,17]. The Tunisian legislation on salt iodization was elaborated, assuming that the per capita daily requirement of iodine is 200 µg and the per capita salt consumption is 10 g per day. The level of iodine required was fixed at 20 mg/kg [41], adding compensation for transit and storage losses of 20% [16]. Thus, the level of iodization required was 24 mg/kg of iodine. Compulsory level of iodine content in the salt was set at 21–27 mg/kg at the producer level and 15–27 mg/kg at the retail level. The goal was to reach a proportion of adequately iodized household salt >90% according to WHO recommendations [4]. As shown, at the national level, and according to the Tunisian legislation, the median of iodine content in salt was in the target range. However, due to a high variability at the national level, about half of households used non-iodized, inadequately, or excessively iodized salt. Thus, the objective of obtaining the “IDD free country certification” of WHO has not yet been achieved in Tunisia.

As also found in other contexts, failures in the technological process during salt iodization [42] and/or post-production losses [43] may account for the inhomogeneous distribution of iodine in the salt commercialized in Tunisia. For example, at the production level, anti-caking agents are essentially to ensure free-flowing salt particles and a sufficient mixing step is needed for a sufficiently homogenous product [41]. The 1996 legislation listed a number of specific dispositions to render compulsory internal quality controls of the salt iodine content, as well as specific instructions regarding packaging. Additionally, legislation was passed in 2005 to create a specific committee devoted to the monitoring of IDD [44]. Thus, in 2000, based on a coverage of 97% of the households using adequately iodized salt, Tunisia was declared IDD free [18]. However, our results would seem to highlight that Tunisian officials have failed to maintain an effective USI program in the long run. This also underlines the need for setting up an effective monitoring system and better coordination of all services involved, which currently are scattered between ministries of health, trade and industry [44].

Regular monitoring of iodized salt production lines must be strengthened with the involvement of producers. Awareness-raising campaigns for the public, producers, and retailers have to be implemented in order to promote iodized salt production and conservation procedures [45].

### 4.6. Strengths and Limitations of the Study

Our study is the first large scale national assessment of iodine status at a population level in North Africa, and one of the few in the MENA region over the last two decades. Due to the high (99.4%) enrollment rate of children in primary school in Tunisia [46], using school children as the sampling base for our large random cluster sample provided adequate coverage of the standard 6–12-year target age class. Nevertheless, there is increasing evidence that inferring the iodine status of populations, based only on data from school-age children, may not be sufficient: Other vulnerable groups, such as pregnant and lactating women, should also be included in monitoring programs [2]. A strength of the study was also that our adequacy assessment (without a formal baseline study and/or control group), included, nevertheless, both impact and performance indicators [5]. The method used for UIC quantification in our laboratory was validated against a reference method (ICP-MS) with which there was good agreement. Beyond assessment of iodine at the population level, based on reference values for medians of UIC, we assessed prevalence of deficiency and excess of iodine by classifying subjects based on the same cutoffs proposed for the median UIC of populations. Although UIC is the recommended measurement for assessment of iodine status at the population level, a limitation is that a single spot measurement may not lead to an accurate assessment of long-term iodine status at the individual level [47]: Indeed, high variability exists in iodine concentrations, so that it would only reflect the ingested iodine during, approximately, the five last hours [48,49]. Thus, the application of the cutoffs to a single spot measurement of UIC may have limitations to classify children into the correct iodine status group [50]. However, the use of a large sample size (from 100 to 500 participants per sub-groups) may compensate for the bias related to the use of only one casual urine sample [51].

## 5. Conclusions

In the framework of a worldwide strategy for eliminating iodine deficiency, Tunisia, a typical middle-income country of the MENA region, embraced the USI approach two decades ago. It passed legislation rendering compulsory iodization of salt commercialized in the country, and, nowadays, aims at being certified an IDD free country by WHO. Our adequacy assessment of the Tunisian USI program showed that, regarding the UIC impact indicator, the program achieved its objectives: ID national rates are now well below the target criteria of WHO certification (though with important geographic disparities). On the other hand, our study underlined that the coverage of households by adequately iodized salt, falls short of the target of certification. This inadequacy, due to a large variability of salt iodine content, also has adverse consequences, in that a non-negligible proportion of the population features an excess of iodine. There are also regional and socio-economic inequalities regarding either deficiency or excess UIC, as well as use of adequately iodized salt. This underlines that, if legislation is a crucial step at the root of health and nutrition policies, evaluation and regular monitoring is vital. Regarding the specific issue of the Tunisian USI program, a review of the national platform and monitoring process could be recommended, aiming towards more coordination of all the institutions involved, as well as encouraging public private partnership. In addition, Tunisia has launched a national strategy to curb the progression of obesity and non communicable diseases, including reducing salt intake, which must be reconciled with the USI program. Lessons learned on how to mitigate the adverse effects of the nutrition transition and preventing IDD will be of interest to many countries in the region that also face this double challenge.

## Figures and Tables

**Table 1:** Socio-demographic characteristics and place of residence of 6–12-year-old Tunisian school children in 2012 (*n* = 1560).

	*n*	% ^1^
**Gender**		
Male	780	51.8
Female	780	48.2
**Age**		
6-7 years	441	28.0
8-9 years	537	37.8
9-12 years	582	37.2
**Milieu**		
Urban	858	64.5
Rural	702	35.5
**Region**		
Greater Tunis	240	19.6
North East	180	14.4
North West	360	11.4
Centre East	240	23.8
Centre West	180	15.6
South East	180	9.7
South West	180	5.6
**Education of the father**		
No formal schooling	92	5.4
Primary schooling	648	40.2
Secondary and more	820	54.4
**Education of the mother**		
No formal schooling	236	13.8
Primary schooling	648	38.6
Secondary and more	676	47.6
**Occupation of the father**		
Not working	93	5.7
Worker/employee	1104	70.1
Middle/upper executive	363	24.2
**Occupation of the mother**		
Not working	1234	74.8
Worker/employee	181	15.3
Middle/upper executive	145	9.9

^1^ Weighted proportions accounting for sampling design.

**Table 2:** Urinary iodine concentration of Tunisian school children aged 6–12 years, by gender, age, milieu, and region.

Variable	Median ^1^	Interquartile Range ^2^
**Gender**		
Boys	241	159–319
girls	200	136–279
**Age**		
6–7 years	228	136–312
8–9 years	213	142–298
9–12 years	221	153–296
**Milieu**		
Urban	225	154–304
Rural	213	140–295
**Region**		
Greater Tunis	228	155–310
North East	159	99–232
North West	213	149–285
Central East	200	140–266
Central West	236	157–295
South East	339	258–430
South West	271	200–347

^1^ Weighted median; ^2^ weighted interquartile range (25th–75th).

**Table 3:** Association between urinary iodine concentration and place of residence, socio-demographic factors, anthropometry and salt iodine content.

		**Unadjusted**						**Adjusted**			
		UIC ^1^ < 100 µg/L			UIC ^1^ ≥ 300 µg/L			UIC ^1^ < 100 µg/L		UIC ^1^ ≥ 300 µg/L	
	*n*	% ^2^	RPR ^3^	C.I. ^5^	% ^2^	RPR ^4^	C.I. ^5^	RPR^3^	C.I. ^5^	RPR ^4^	C.I. ^5^
**Gender**											
		*p* ^6^ = 0.44			*p* ^7^ < 0.001			*p* ^6^ = 0.41		*p* ^7^ < 0.001	
Male	780	9.9%	1	–	30.1%	1	–	1	–	1	–
Female	780	13.1%	1.2	0.8–1.8	19.6%	0.6	0.4–0.8	1.2	0.8–1.8	0.5	0.3–0.7
**Age**											
		*p* ^6^ = 0.06			*p* ^7^ = 0.54			*p* ^6^ = 0.045		*p* ^7^ = 0.41	
6–7 years	441	13.4%	1	–	26.9%	1	–	1	–	1	–
8–9 years	537	12.7%	0.9	0.6–1.4	23.9%	0.8	0.6–1.2	0.9	0.6–1.6	0.8	0.6–1.2
9–12 years	582	8.7%	0.6	0.4–0.9	24.8%	0.8	0.6–1.2	0.6	0.4–0.9	0.8	0.6–1.1
**Milieu**											
		*p* ^6^ = 0.92			*p* ^7^ = 0.75			*p* ^6^ = 0.89		*p* ^7^ = 0.89	
Urban	858	11.4%	1.0	0.5–2.0	25.7%	1.1	0.6–2.2	1.0	0.5–1.8	1.0	0.6–1.5
Rural	702	11.4%	1	–	23.8%	1	–	1	–	1	–
**Region**											
		*p* ^6^ < 0.001			*p* ^8^ < 0.001			*p* ^6^ = 0.008		*p* ^7^ < 0.001	
Greater Tunis	240	11.2%	1	–	28.1%	1	–	1	–	1	–
North East	180	25.5%	2.3	1.5–3.4	13.1%	0.5	0.2–1.2	2.1	1.4–3.1	0.4	0.2–1.1
North West	360	8.4%	0.7	0.4–1.2	22.1%	0.7	0.2–2.0	0.6	0.3–1.2	0.6	0.2–1.8
Central East	240	8.9%	0.6	0.3–1.4	15.2%	0.4	0.1–1.4	0.6	0.3–1.3	0.4	0.1–1.4
Central West	180	9.7%	0.8	0.3–2.2	22.8%	0.7	0.2–2.4	0.8	0.3–1.9	0.6	0.2–1.9
South East	180	7.5%	1.4	0.3–6.2	62.5%	4.5	1.5–13.9	1.2	0.3–5.2	4.9	1.6–14.7
South West	180	4.3%	0.4	0.2–0.9	34.2%	1.2	0.4–3.4	0.4	0.1–1.0	1.1	0.4–3.0
**Education of the father**											
		*p* ^6^ = 0.54			*p* ^7^ = 0.16			*p* ^6^ = 0.26		*p* ^7^ = 0.032	
No formal schooling	92	9.8%	0.9	0.4–1.7	32.7%	1.5	1–2.3	0.9	0.4–1.9	1.8	1.2–2.7
Primary schooling	648	9.9%	0.8	0.5–1.2	25.8%	1.1	0.8–1.5	0.7	0.4–1.2	1.2	0.9–1.8
Secondary and more	820	12.7%	1	–	23.8%	1	–	1	–	1	–
**Education of the mother**											
		*p* ^6^ = 0.17			*p* ^7^ = 0.77			*p* ^6^ = 0.41		*p* ^8^ = 0.52	
No formal schooling	236	6.8%	0.6	0.3–1.2	29.1%	1.2	0.7–2.1	0.7	0.3–1.7	1.4	0.8–2.5
Primary schooling	648	12.6%	1.1	0.6–2.0	24.6%	1.0	0.7–1.4	1.2	0.7–2.1	1.1	0.8–1.6
Secondary and more	676	11.7%	1	–	24.3%	1	–	1	–	1	–
**Occupation of the father**											
		*p* ^6^ = 0.24			*p* ^7^ = 0.79			*p* ^6^ = 0.033		*p* ^7^ = 0.82	
Not working	93	13.3%	1.5	0.8–2.9	22.9%	1.0	0.4–2.3	1.9	1.0–3.5	1.0	0.4–1.8
Worker/employee	1104	12.1%	1.4	0.9–2.2	25.3%	1.1	0.8–1.4	1.7	1.1–2.5	0.8	0.4–1.7
Middle/upper executive	363	9.0%	1	–	24.8%	1	–	1	–	1	–
**Occupation of the mother**											
		*p* ^6^ = 0.86			*p* ^7^ = 0.88			*p* ^6^ = 0.75		*p* ^7^ = 0.45	
Not working	1234	11.6%	0.9	0.5–1.6	24.8%	0.9	0.5–1.5	0.7	0.3–1.9	0.9	0.4–2.0
Worker/employee	181	9.9%	0.8	0.3–2.1	24.9%	0.9	0.4–1.7	0.9	0.5–1.6	0.7	0.4–1.3
Middle/upper executive	145	12.1%	1	–	26.9%	1	–	1	–	1	–
**Body Mass Index forage (z–scores)**											
		*p* ^6^ = 0.66			*p* ^8^ = 0.29			*p* ^6^ = 0.76		*p* ^8^ = 0.15	
Thinness(<–2)	138	9.5%	0.8	0.4–1.8	23.7%	0.9	0.5–1.6	1.0	0.4–2.1	1.0	0.6–1.7
Normal weight (–2 to <+1)	1162	11.2%	1	–	24.7%	1	–	1	–	1	–
Overweight (≥+1)	260	13.7%	1.4	0.6–3.0	27.2%	1.2	0.9–1.7	1.4	0.6–3.3	1.4	0.9–2.1
**Iodine content of salt used in the household (mg/kg)**											
		*p* ^6^ = 0.24			*p* ^7^< 0.001			*p* ^6^ = 0.22		*p* ^7^ = 0.002	
*Non iodized (0)*	103	7.6%	0.6	0.3–1.5	16.5%	0.5	0.3–0.9	0.7	0.3–2.0	0.5	0.2–1.0
*Inadequately iodized (>0* to *<15)*	207	17.0%	1.5	1.0–2.5	15.6%	0.5	0.4–0.8	1.5	1.0–2.3	0.7	0.4–1.1
*Adequately iodized(≥15* to *<25)*	890	10.4%	1	–	26.0%	1	–	1	–	1	–
*Excessively iodized (≥25)*	360	10.8%	1.1	0.7–1.9	29.8%	1.0	0.8–1.4	1.0	0.6–1.7	1.1	0.8–1.5

^1^ UIC: Urinary iodine concentration; ^2^ Weighted % (accounting for sampling design, including unequal probabilities of selection); ^3^ RPR: For category of cofactor vs. reference category (for which RPR = 1), crude or adjusted Relative Prevalence Ratio of having UIC < 100 vs. having UIC in the 100 < UIC < 300 category (base response category); ^4^ RPR: For category of cofactor vs. reference category (for which RPR = 1), crude or adjusted Relative Prevalence Ratio of having high iodine (IUC ≥ 300) vs. having UIC in the 100 < UIC < 300 category (base response category); ^5^ C.I.: 0.95 sampling design based confidence interval for crude or adjusted RPR; ^6^ Crude or adjusted P-value for association of ID (iodine <100) with co-factor; ^7^ Crude or adjusted *p*-value for association of excess of iodine (≥300) with a co-factor.
